# Accuracy of Computed Tomography in Differentiating Perforated from Nonperforated Appendicitis, Taking Histopathology as the Gold Standard

**DOI:** 10.7759/cureus.3735

**Published:** 2018-12-15

**Authors:** Mohammad Ali, Jawaid Iqbal, Raza Sayani

**Affiliations:** 1 Radiology, Dow University, Karachi, PAK; 2 Radiology, Liaquat National Hospital, Karachi, PAK; 3 Radiology, Aga Khan University, Karachi, PAK

**Keywords:** acute appendicitis, computed tomography, non-perforated appendicitis

## Abstract

Introduction

Acute appendicitis is one of the more common causes of acute abdominal pain. It occurs when the lumen of the appendix is obstructed, leading to inflammation and finally perforation. The preoperative differentiation of perforated from nonperforated appendicitis is important and helpful to define prognosis and determine an adequate therapeutic approach, including consideration for nonsurgical treatment. This study recommends computed tomography (CT), a noninvasive method of investigation, be used frequently in clinically suspected cases of perforated appendicitis in the Pakistani population for better patient outcomes.

Objective

To determine the diagnostic accuracy of CT in differentiating perforated from nonperforated appendicitis by using histopathology as the gold standard.

Material and methods

A total of 236 patients with a clinical suspicion of appendicitis were included in this study. CT was performed in Liaquat National Hospital and Medical College. At the time of scanning, intravenous contrast was administered. Histopathology was used as the diagnostic gold standard. CT findings were documented using a proforma. The patient was returned to the referring department and followed after surgery for histopathology.

Results

Sensitivity, specificity, and positive and negative predictive values, as well as the accuracy of CT in the detection of perforated appendicitis, was 71.4%, 90.7%, 62.5%, 93.6%, and 87.3%, respectively.

Conclusion

CT findings can be used to select patients with perforated appendicitis for initial nonoperative management.

## Introduction

Acute appendicitis is one of the more common causes of acute abdominal pain, with an incidence of 33.8 cases per 100,000 individuals per year, with a lifetime risk of 9%. The incidence of appendicitis has increased in recent years at an average rate of 0.5 cases per 100,000 individuals per year [1].

Appendicitis occurs when the lumen of the appendix is obstructed, leading to inflammation and finally perforation. Individuals from Hispanic and Asian origins have higher rates of perforation [1]. The incidence of perforated appendicitis is high in developing countries like Pakistan, with approximately 20% of appendicitis cases being perforated [2].

Preoperative differentiation of perforated from nonperforated appendicitis is important and helpful to define prognosis and determine an adequate therapeutic approach, including consideration for nonsurgical treatment. The incidence rate of postoperative complications in patients with perforated appendectomy is high compared to nonperforated appendectomy (28.4% vs. 4.7%) [3]. In one study, the mean length of hospital stay in the perforated group was 6.3 days while it was 2.9 days in the nonperforated group [4].

In this modern era of imaging, computed tomography (CT) has played a vital role in diagnosing appendicitis and in differentiating perforated from nonperforated appendicitis. It is noninvasive, compared to invasive methods like diagnostic laparoscopy [5-11]. In one study, the sensitivity and specificity of CT to diagnose perforated appendicitis was 69% and 97%, respectively [4].

The purpose of this study is to evaluate the usefulness of the noninvasive modality CT in differentiating perforated from nonperforated appendicitis and define its sensitivity and specificity in differentiating patients with perforated appendicitis from those with nonperforated appendicitis. Histopathology was used as the diagnostic gold standard.

The rates of appendectomy in patients with perforated appendicitis have decreased since 1995 [1]. If perforated appendicitis is diagnosed preoperatively, the management of the patient is different, as percutaneous drainage followed by interval appendectomy is recommended for patients with perforated appendicitis [1]. On the other hand, simple laparoscopic appendectomy is needed for patients with nonperforated appendicitis; therefore, it is important to diagnose perforated appendicitis preoperatively.

There is not enough or recent local data on this topic; therefore, the purpose of this study is to evaluate the accuracy of the noninvasive modality CT in differentiating perforated from nonperforated appendicitis for the local population.

## Materials and methods

We conducted this cross-sectional study at the department of radiology at Liaquat National Hospital and Medical College for six months. A total of 236 patients with clinical suspicion of appendicitis and aged 15 to 70 years were included. These patients underwent surgery within a week of their CT scan. Patients who were refused surgery, referred to other hospitals, or with deranged renal function were excluded.

Informed consent was obtained prior to the procedure. CT was performed on a Toshiba Activion™ 16 Multislice CT scanner (Toshiba Medical Systems Corp., Tokyo, Japan). The scanning protocol included the acquisition of axial helical CT sections before and after the administration of intravenous contrast, extending from the xiphoid process of the sternum to the pubic symphysis at 120-kVp and 210 mA. At the time of scanning, intravenous contrast was administered using a power injector at a rate of 5 mL per second followed by the acquisition of axial cuts at 4-mm slice thickness in the portal venous phase (60 to 70 seconds after injection of bolus contrast). Sagittal and coronal multiplanar reconstruction was also performed. CT scans were interpreted by consultant radiologists with a minimum of five years of experience. Histopathology was used as the diagnostic gold standard. CT scan findings were documented by the researcher using a proforma. Data were analyzed on Statistical Package for Social Sciences (SPSS) Statistics for Windows, Version 17.0 (SPSS Inc., Chicago, IL, US). Relevant descriptive statistics, frequency, and percentage were computed for gender, CT scan findings, and histopathology findings. Mean ± standard deviation for age were calculated. Sensitivity, specificity, positive and negative predictive values, and diagnostic accuracy of perforated appendicitis on CT scan were calculated using histopathology as the gold standard. Stratification was performed to control effect modifiers like age and gender to observe the effect of these modifiers on the accuracy through chi-square test; p<0.05 was considered significant.

## Results

A total of 236 patients with a clinical suspicion of appendicitis were included in this study. The average age of the patients was 40 ± 13 years; 144 (61.02%) were male and 92 (38.98%) female.

**Figure 1:**
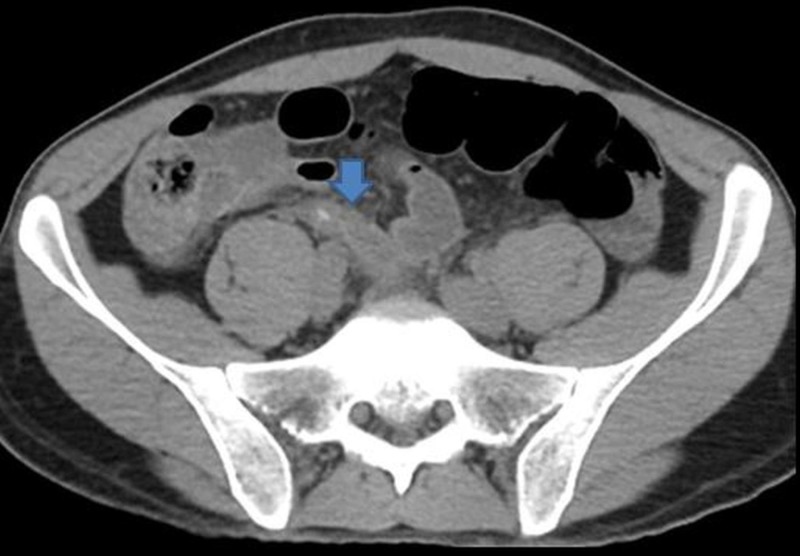
Acute appendicitis without perforation Axial image without contrast shows the swollen appendix with appendicolith (arrow). Periappendiceal inflammatory changes seen.

The following, seen in approximately 25% of patients, were taken as specific signs of nonperforated appendicitis on CT scan: enlarged appendiceal diameter (> 6 mm) with an occluded lumen; appendiceal wall thickening (> 2 mm); periappendiceal fat stranding; appendiceal wall enhancement; and appendicolith (Figure 1).

**Figure 2:**
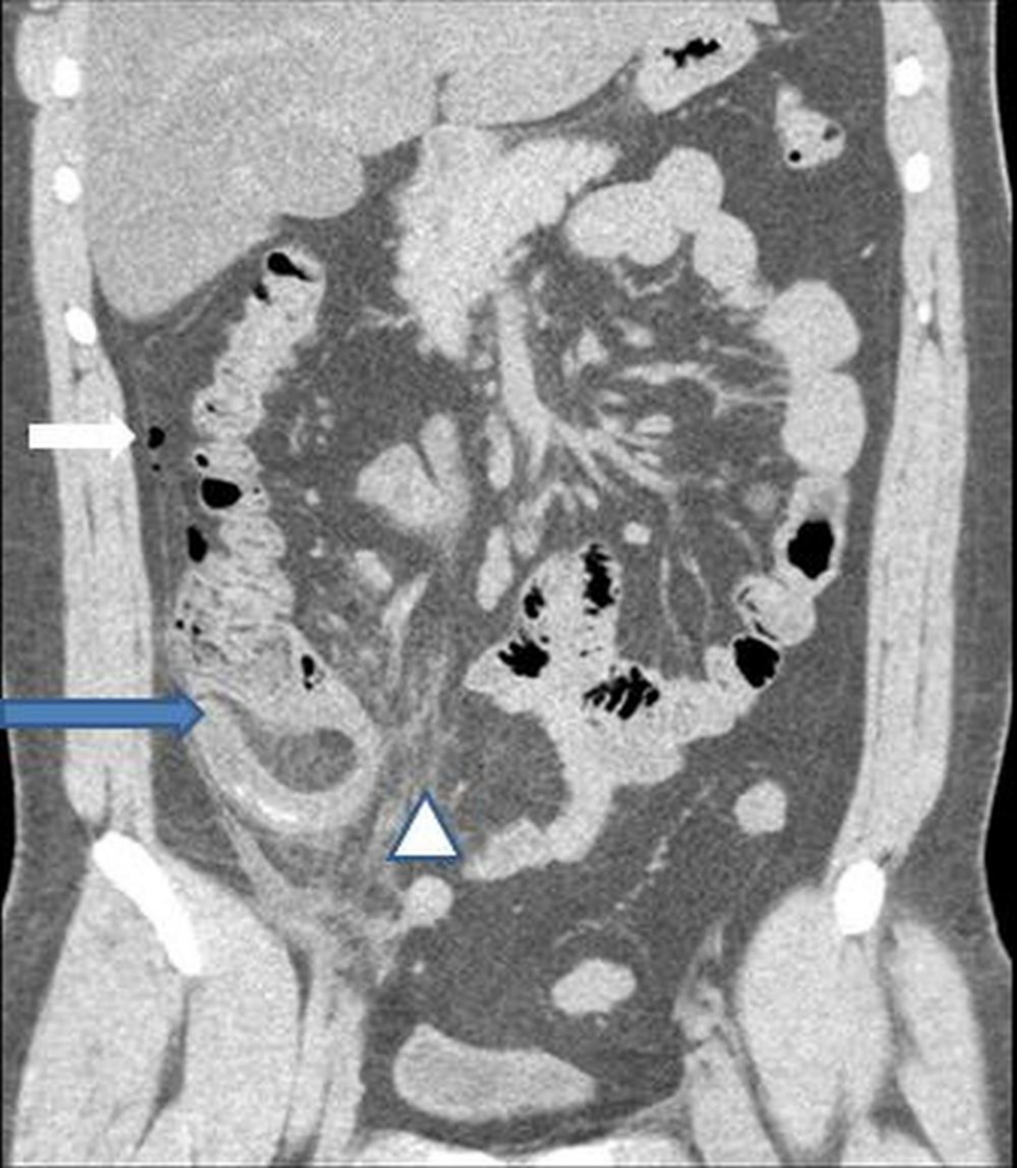
Coronal image of abdomen showing swollen appendix (blue arrow) with marked periappendiceal inflammatory fat stranding (arrowhead). Small amount of free air also seen (white arrow)

**Figure 3:**
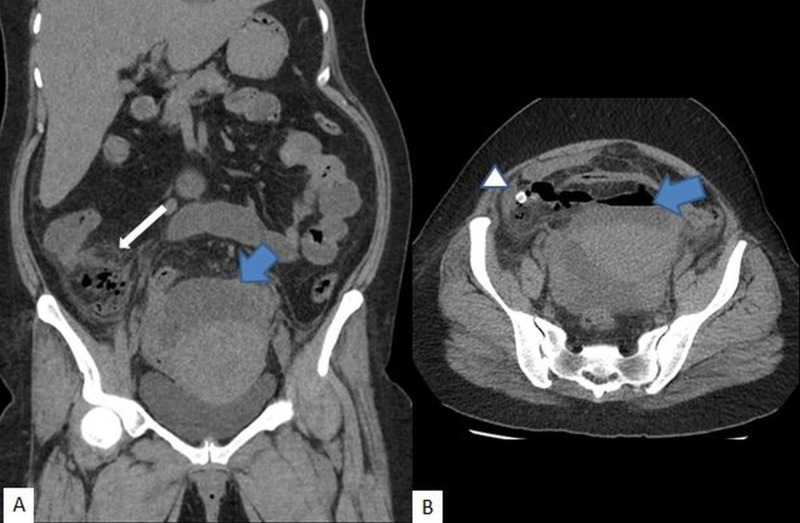
Acute appendicitis with perforation A) Coronal and B) axial images demonstrated collection (blue arrow), inflammatory changes in the right iliac fossa (white arrow); appendicolith (arrowhead)

Specific signs for perforated appendicitis on CT scan included a defect in enhancing the appendiceal wall, focal area of nonenhancement with enhancing of the remaining appendiceal wall, extraluminal air, and extraluminal appendicolith or abscess formation (Figures 2-3).

CT reported that there were 20.34% (48/236) cases that had perforated and 79.66% had non-perforated appendicitis while histopathology reported 17.8% (42/236) with perforated and 82.2% with non-perforated appendicitis.

Sensitivity, specificity, positive and negative predictive values, as well as the accuracy of CT in the detection of perforated appendicitis, was 71.4%, 90.7%, 62.5%, 93.6%, and 87.3% respectively.

The accuracy of CT in the detection of perforated appendicitis was 85.4% in male cases and 92.2% in female cases. Similarly, concerning age groups, the accuracy of CT was above 92% for patients older than 40 years and 82.9% for younger or equal to 40 years.

## Discussion

Opting for nonsurgical management in cases of perforated appendicitis depends on accurate and reliable CT interpretation. Sensitivities and specificities for CT for the diagnosis of acute appendicitis are around 90%, resulting in significantly reduced negative appendectomy rates from 15%–20% to 2%–12% [12-16]. Despite this high sensitivity, the differentiation between perforated and nonperforated appendicitis is as accurate as it should be.

Distinguishing perforated from nonperforated appendicitis depends on a wide range of features as seen on CT, which include the presence of free fluid, phlegmon, abscess, extraluminal air, and bowel wall thickening; each of these characteristics favor perforation [6,17-18].

In the present study, the average age of the patients was 40 ± 13 years. Of 236 cases, 144 (61.02%) were male and 92 (38.98%) female. Similarly, in the Kim et al. study [19], there were 339 patients with a mean age of 40.8 years (range, 19 to 80 years); 183 were male (mean age, 40.5 years; range, 19 to 79 years) and 156 were female (mean age, 41.2 years; range, 19 to 80 years).

In our study, sensitivity, specificity, positive and negative predictive values, as well as the accuracy of CT in the detection of perforated appendicitis, were 71.4%, 90.7%, 62.5%, 93.6%, and 87.3%, respectively. In another study, the sensitivity and specificity of CT to diagnose perforated appendicitis was 69% and 97%, respectively [4]. In the Fraser et al. study [9], CT had a sensitivity of 62% with a specificity of 81% in predicting appendiceal perforation.

One retrospective study found that using a defect in the enhancing appendiceal wall as the sole CT finding to determine perforation increased the sensitivity, specificity, and accuracy to 95.0%, 96.8%, and 96.1%, respectively [8].

In this study, the accuracy of CT in the detection of perforated appendicitis was 85.4% in male cases and 92.2% in female cases. Similarly, concerning age groups, the accuracy of CT was greater than 92% for patients older than 40 years and 82.9% for those younger to or equal to 40 years. Despite the existence of many studies on CT diagnosis of appendicitis, to our knowledge, few have focused on the differential diagnosis of perforated vs. nonperforated appendicitis. A study by Horrow et al. involved single-section helical CT with several scanning protocols. Specifically, the section thickness ranged from 5 mm to 10 mm [6]. In addition, some examinations were performed with oral contrast material while others were performed with intravenous contrast material. In that study, a defect of the enhancing appendiceal wall was most sensitive as a single finding, but its sensitivity remained at 64%. On the other hand, a combination of four findings (abscess, phlegmon, extraluminal air, and extraluminal appendicolith) had higher diagnostic accuracy. However, the use of a combination of several findings may be complicated in an emergency. Another study [20] based specificity on a focal defect of the enhancing appendiceal wall.

Our study was limited in that it is a single-center study with a relatively small patient population. Also, the interpretation of CT scans was performed by several radiologists with a variety of experience levels.

## Conclusions

A CT scan is markedly sensitive, as well as specific, for the differentiation of perforated from non-perforated appendicitis. This, in turn, helps in patient selection for initial nonoperative management.
